# Augmented Reality in Spine Surgery: A Narrative Review of Clinical Accuracy, Workflow Efficiency, and Barriers to Adoption

**DOI:** 10.7759/cureus.86803

**Published:** 2025-06-26

**Authors:** Ahmed Nadeem-Tariq, Sarah Kazemeini, Pratiksha Kaur, Grace Dang, Trevor Davis, Kiratpreet Sraa, Philip Zitser, Christopher Fang

**Affiliations:** 1 Otolaryngology - Head and Neck Surgery, University of Nevada, Las Vegas (UNLV) School of Medicine, Las Vegas, USA; 2 Medical Education, University of Nevada, Las Vegas (UNLV) School of Medicine, Las Vegas, USA; 3 Dermatology, Stony Brook University, Stony Brook, USA; 4 Dermatology, University of Missouri–Kansas City School of Medicine, Kansas City, USA; 5 Dermatology, West Virginia School of Osteopathic Medicine, Lewisburg, USA; 6 Dermatology, Midwestern University Arizona College of Osteopathic Medicine, Glendale, USA; 7 Dermatology, New York Institute of Technology College of Osteopathic Medicine, Old Westbury, USA; 8 Orthopedics, Kirk Kerkorian School of Medicine at University of Nevada, Las Vegas (UNLV), Las Vegas, USA

**Keywords:** augmented reality, orthopedic, orthopedic surgery, virtual reality, virtual reality simulation

## Abstract

Augmented reality (AR) has emerged as a promising intraoperative navigation tool in spine surgery, particularly for pedicle screw placement, which demands high spatial precision and carries risks of neurovascular injury. AR platforms offer real-time overlays of patient anatomy, aiming to enhance accuracy, reduce radiation exposure, and streamline operative workflow. This narrative review synthesizes the early clinical experiences, efficacy data, and barriers to adoption for AR-assisted navigation in spine surgery. It also explores the role of AR in surgical training, workflow optimization, and future integration into routine practice. A comprehensive search of PubMed and ScienceDirect was conducted for English-language studies from January 2004 to May 2025. Inclusion criteria focused on peer-reviewed trials evaluating AR in spinal procedures, with outcomes including screw placement accuracy, radiation exposure, operative time, and user experience. Across multiple platforms, including Microsoft HoloLens and Augmedics xVision, AR-assisted navigation demonstrated pedicle screw placement accuracy rates ranging from 93% to 100%, often with reduced fluoroscopy time and improved surgeon ergonomics. Preliminary data suggest utility in complex deformity surgeries and surgical training. However, widespread adoption remains limited by cost, integration challenges, and a lack of large-scale, multicenter trials. AR represents a viable adjunct to current navigation technologies in spine surgery, showing early promise in enhancing precision and efficiency. Broader adoption will require improved validation, standardization of training protocols, and cost-effectiveness data.

## Introduction and background

Overview of augmented reality (AR) in surgery

AR refers to a group of technologies that superimpose digital information, such as three-dimensional anatomical models or instrument trajectories, over the real-world surgical field in real time. Unlike virtual reality, which immerses you in an artificial environment, AR improves a surgeon's natural viewpoint by overlaying contextually relevant data via head-mounted displays (HMDs), smart glasses, or see-through monitors with spatial tracking capabilities [1]. This live overlay allows the surgeon to maintain visual contact with the operating field while requiring minimal cognitive effort. AR is fundamentally different from existing surgical navigation systems, which rely on external screens located far from the operation region, necessitating clinicians to shift their attention and mentally reconstruct spatial information [2]. AR aims to simplify procedures that require high spatial precision. AR, particularly in surgeries that require high spatial accuracy, aims to simplify intraoperative decision-making by directly integrating anatomical landmarks and surgical plans into the surgeon's field of vision. AR's use in surgery has increased in recent years, owing to technological advancements and a growing demand for precision-enhancing technologies in minimally invasive and complex anatomical contexts [3]. These advancements have paved the way for the use of AR in high-risk surgical specialties, such as orthopedic spine surgery.

Clinical context of spine surgery

Because key neurovascular systems are closely located, spine surgery, particularly pedicle screw implantation, requires millimeter-level accuracy. Pedicle screw placement is a precise procedure; even a few degrees off-angle can cause nerve damage, hardware failure, or necessitate a return to the operating room [4]. Navigation methods have proven useful, but many have drawbacks, including radiation exposure from fluoroscopy, poor depth perception from 2D displays, and the challenging ergonomics of robotic arms, not to mention the steep learning curve. Augmedics xVision and Microsoft HoloLens are two AR solutions that overcome these limitations by providing real-time overlays of patient anatomy, screw trajectories, and virtual markers within the surgeon's visual field [5]. These solutions are designed to maintain intuitive hand-eye coordination while enhancing accuracy and minimizing workflow interruptions. Early clinical data indicate that AR can produce equivalent or superior results to conventional systems, particularly in minimally invasive settings where spatial orientation is fundamentally difficult [6]. AR technology has the potential to improve visual guiding during spinal instrumentation procedures.

Background and clinical need

**Figure 1:**
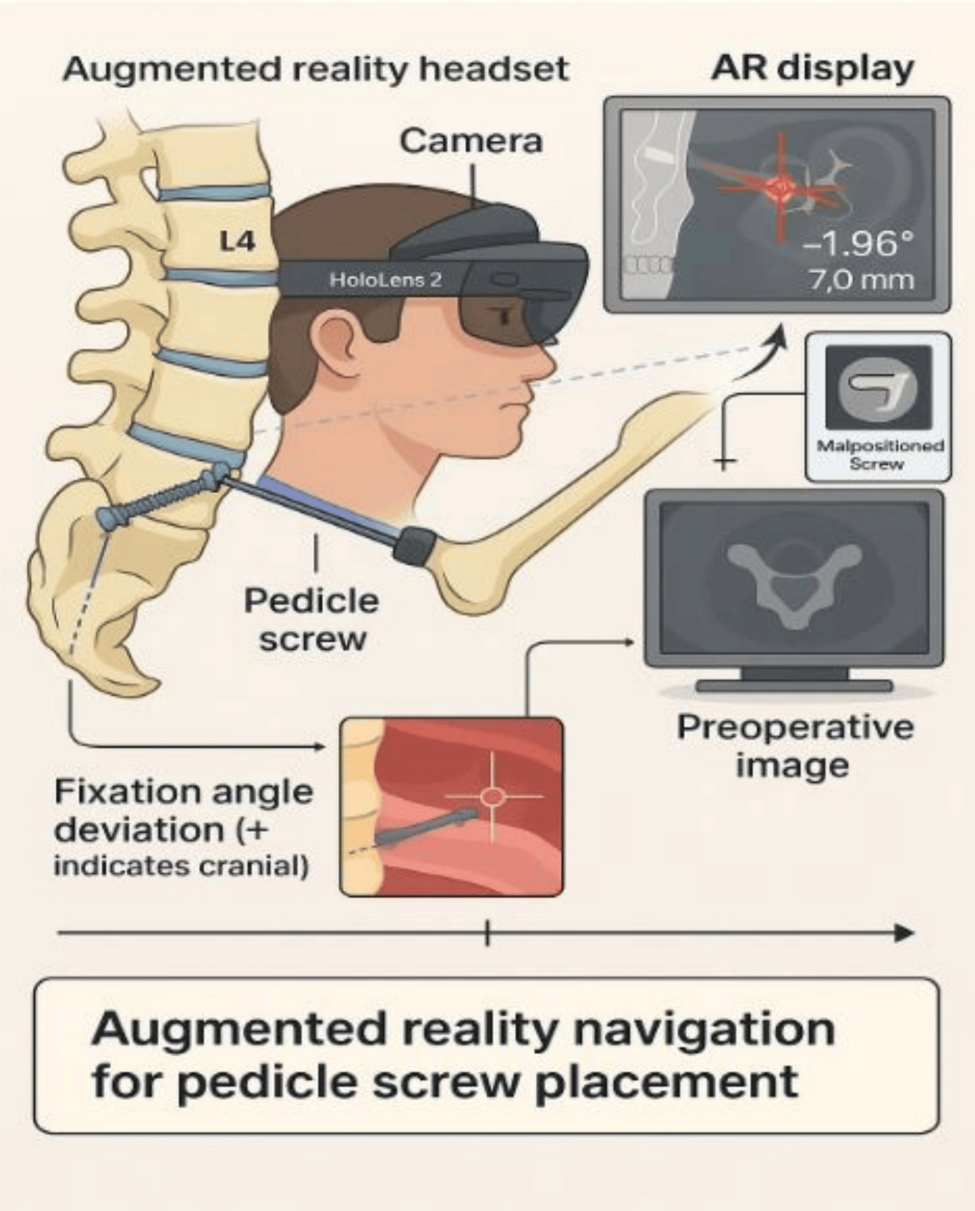
Workflow of augmented reality-assisted pedicle screw placement using a head-mounted display (HoloLens 2). Real-time visualization of screw trajectory is overlaid onto the surgical field using intraoperative data. The system provides guidance on fixation angle deviation and potential malposition warnings, enhancing accuracy while minimizing attention shifts and radiation exposure. The image was created by the research team using BioRender (https://www.biorender.com/).

The correct placement of pedicle screws is crucial for achieving spinal stability, preserving neurologic function, and minimizing the need for revision surgery. Malpositioned screws occur in 10-15% of freehand cases, with higher rates seen in anatomically difficult or minimally invasive procedures [7]. Misplaced screws may result in complications such as dural tears, nerve root compression, and spinal instability. Traditional techniques for improving screw precision, such as intraoperative fluoroscopy or CT-based navigation, have reduced complications, but they are associated with longer operative times and increased radiation exposure for both patients and surgical personnel [8]. Furthermore, these technologies frequently require surgeons to shift their attention between the patient and an external monitor, disrupting natural hand-eye coordination and lengthening the operative time [9]. Some platforms also require expensive and complex infrastructure, which is not readily available in many surgical facilities. AR aims to address these limitations by incorporating navigation into the visual field, potentially improving safety, accuracy, and surgical efficiency without requiring multiple fluoroscopic confirmations. As AR systems advance, their role in altering spine surgical navigation may become more important, especially in high-risk or resource-constrained settings. Figure 1 provides a good layout of what is expected through the integration of AR in the operating room.

Objective and scope of the review

Aim of the Literature Review

AR is a novel assistive technology that integrates data visualization to improve navigation in spine surgery. The accuracy of AR-assisted navigation in pedicle screw placement has been well documented [10]. Pedicle screw placement has been correlated with complication rates, as they are located near pivotal neural and vascular structures. Methods such as using anatomic landmarks and fluoroscopic or computed tomography-guided imaging have been employed to ensure safety when navigating complex structures. On the downside, employing these methods results in notable radiation exposure, prolonged preoperative time, and proves to be expensive. AR-assisted navigation in spine surgery serves as a promising alternative method as it improves visualization of the surgical field with 3D intraoperative imaging generated by optical cameras [10]. A total of 20 studies reported clinically accurate placement of screws, demonstrating its reliability. The accuracy of AR-assisted navigation in spine surgery was corroborated by Liu et al. (2022), who conducted a systematic literature review to assess the status of AR-assisted navigation in spinal surgery. Through Liu et al.'s systematic review, it was demonstrated that AR-assisted navigation, a non-invasive adhesive skin marker, can significantly prevent loss of tracking due to accidental patient movement, as it is not impacted by respiration [11].

Additionally, the AR display of spatial position facilitates visual analysis, enhancing the accuracy of surgery by providing a general pathway that surgeons can follow. Additionally, AR also provides surgeons with concurrent spatial views that enhance the precision of pedicle screw placement in spinal surgery. Not only improving intraoperative navigation but also potentially improving postoperative outcomes with decreased operative time, blood loss, and better clinical outcomes [12]. In addition to the efficiency of AR-assisted navigation in terms of spatial display and operative workflow, it is also important to consider its implications for user experience. According to Wolf et al. (2023), a study was conducted in which AR visualizations displaying the drill trajectory through Microsoft HoloLens were used by four expert surgeons and 10 novice surgeons to assess user experience and trajectory deviation. Wolf et al. revealed that participants, regardless of expertise status, successfully placed pedicle screws within a 2° trajectory deviation. Both the expert and novice surgeons indicated that the AR's peripheral rings allowed them to stay focused on the area of execution, further easing cognitive load [13]. Overall, the current literature emphasizes the effectiveness of AR-assisted navigation in spine surgery, particularly in pedicle screw placement, which has been shown to decrease cognitive load for users and reduce preoperative time.

Broader Goals and Barriers

Integration in complex surgeries: The current literature emphasizes the use of AR-assisted navigation in standard spinal procedures; however, its integration into complex surgeries and procedures presents limitations and complexities. AR-assisted navigation can achieve accuracy ranging from 93.0% to 98.5% [14], therefore reducing the risk of neurological and vascular complications in patients presenting with severe spinal deformities, as this technology enhances the surgeon's ability to precisely and accurately place the pedicle screw. For instance, Chang et al. (2025) conducted a retrospective observational study that included 10 patients who underwent surgery for thoracolumbar scoliosis. This procedure was carried out with AR-assisted navigation, resulting in an overall instrumentation accuracy of 98%. Notably, none of the patients in the cohort developed postoperative neurological complications [14]. It is essential to note that AR-assisted navigation also offers a significant advancement in preoperative consultation, where patients and their families receive clearer and more realistic information about the upcoming surgical procedure. As a result, AR-assisted navigation not only improves the accuracy of pedicle screw placement but also improves the operative workflow. Despite these advancements, many studies similar to Chang et al. employ a relatively small patient cohort, therefore hindering the generalizability of the findings and treatments. Similar outcomes were observed with Sakai et al.'s study, which conducted a prospective controlled clinical trial on patients with adolescent idiopathic scoliosis (AIS) [15]. The study concluded that AR technology not only enhanced spinal correction rates but also improved the well-being and fatigue of surgeons. Moreover, AR-assisted surgeries resulted in an overall improvement in the activity and mood of the surgeons, as well as a reduction in operative time and blood loss compared to standard AIS surgeries. Nevertheless, similar to Chang et al.'s study, Sakai et al.'s study was limited by a small sample size. To be more specific, three surgeons carried out the surgical procedure, all of whom were males; therefore, the beneficial outcomes cannot be generalized to female surgeons, further highlighting the need for studies to employ multicenter data to increase the overall generalizability of findings and treatments.

Adoption barrier

Despite its potential, technical and economic barriers persist in the adoption of AR technologies. As indicated by Ramirez et al. (2024), the use of AR technologies has a learning curve as surgeons navigate to master the system's complexities [16]. Additionally, the incorporation of AR technology necessitates setting aside a significant learning period to introduce the system's complexities to nurses, the medical team, and technical staff. Therefore, the incorporation of AR technologies during the learning stage may extend the surgery time. As a result, experienced surgeons are discouraged from adopting AR technologies in their routine practice [16]. This idea was further corroborated by Butler et al., highlighting that surgeons must learn to seamlessly incorporate various inputs from AR-assisted navigation technologies to enhance performance in spinal surgeries [17]. The implementation of AR technologies is a costly process as the equipment, software development, and maintenance can pose an economic barrier [16]. As a result, it hinders the widespread adoption of AR technologies, particularly in resource-constrained areas.

The need for multicenter data and long-term analysis

While most studies highlight the accuracy of AR-assisted navigation in spinal surgical procedures, many contain a small sample size. Therefore, to validate the current literature, there is a need for studies employing multicenter data, which can increase the sample's diversity and size. As a result, it increases the generalizability of the findings and the treatment. Additionally, the current literature emphasizes that AR technologies decrease postoperative complications [14]; however, many of them fail to consider the long-term impacts on the patient. As a result, there is an increased need for studies that focus on long-term outcomes of patients who have undergone spinal surgery with AR-assisted navigation technologies. Although postoperative outcomes may have improved, examining long-term outcomes represents a crucial component that has not been extensively addressed in the current literature [18].

## Review

Methods

This narrative literature review aims to summarize existing research on the integration and application of AR in spine surgery, including pedicle screw insertion. A comprehensive review was conducted in the PubMed and ScienceDirect databases for peer-reviewed papers published between January 2004 and May 2025. The search technique used keywords and MeSH (Medical Subject Headings) phrases including "augmented reality", "spine surgery", "pedicle screw", "AR navigation", "xVision", and "Microsoft HoloLens". Boolean operators such as "AND" and "OR" were used to narrow the search results. The reference lists of selected publications were also manually reviewed to identify additional relevant research that was not discovered in the database queries.

Studies evaluating the clinical use, practicability, or performance of AR platforms in spine surgery were considered. Eligible trials provided critical criteria, including pedicle screw accuracy, operational efficiency, radiation exposure, surgeon ergonomics, and training effectiveness. Prospective clinical research and observational cohort studies were also included, as were systematic reviews and high-quality cadaveric or simulation trials. Articles were confined to English-language media with full-text access. Preference was given to studies that employed commercially available or confirmed AR technologies, such as the Augmedics xVision platform, Microsoft HoloLens, and microscope-based augmented reality surgical navigation (ARSN) devices [19-21].

The methodological quality of each study was assessed using four criteria: design, sample size, statistical rigor, and repeatability. Control groups were examined, as were randomization and bias avoidance methods. Studies with small patient populations, pilot designs, or observational restrictions were included to offer context but should be interpreted with caution. Common sources of bias were novelty bias toward emerging technology, small sample bias, and performance bias due to a lack of blinding. Where applicable, the evaluation focused on quantitative outcomes, such as Gertzbein-Robbins pedicle screw grading, mean deviation angles, cortical breach rates, and intraoperative fluoroscopy dose reduction. Data were gathered on clinical performance (e.g., screw insertion accuracy), operational parameters (e.g., time, radiation exposure), and user experience [22].

Johnson et al. (2023) and Yahanda et al. (2021) reported high-resolution data on screw accuracy using AR-guided devices, while Sakai et al. [15] and Ramirez et al. [16] investigated the influence of AR on workflow and user fatigue in the treatment of complicated spinal deformities. These results were critically reviewed to identify similar trends in AR performance, as well as significant evidence gaps, particularly in terms of generalizability, cost-effectiveness, and long-term postoperative outcomes. The review was conducted and published in accordance with the academic guidelines for the narrative synthesis of surgical technology literature [23-25].

Clinical outcomes and efficacy

Screw Accuracy and Placement Metrics

The importance of accurate pedicle screw placement not only prevents complications such as damaging surrounding nerves or the need for replacement surgery but also improves patient outcomes by promoting long-term stability. The Gertzbein-Robbins scale is used to assess the clinical acceptability of pedicle screw placements based on the degree of the cortical breach. A study by Gubian et al. (2022) found that all 140 pedicle screws placed using CT navigation were rated grade A (no cortical breach) or B (breach of less than 2 mm) on the GR scale, which are considered clinically acceptable. However, since the screws were preoperatively planned to meet grade A standards, deviations suggest that while the GR scale can provide safety measures, it may not fully capture the nuances in surgical precision (Gubian et al., 2022). The xVision-Spine (XVS) system has been shown to be effective in spine surgery. Johnson et al. (2023) reports a GR accuracy of 97.1% across 218 AR-guided screws, with no intraoperative or early postoperative complications. This system utilizes an HMD AR guidance. Further studies have examined the precision of screw placement using AR technology [26].

Yahanda et al. [23] achieved 100% accuracy in placing 63 percutaneous pedicle screws using AR guidance, with all screws graded as either A or B on the Gertzbein-Robbins scale. Similarly, Elmi-Terander et al. [24] reported a Gertzbein-Robbins accuracy rate of 94.1% across 253 AR-guided screws placed in 20 patients, with minor breaches occurring in patients with scoliosis, but no screws experiencing severe misplacement. Liebmann et al. [25] found that using the Microsoft HoloLens for lumbar pedicle screw placement resulted in a mean error of 2.77±1.46 mm for screw insertion points and 3.38°±1.73° for the screw trajectory orientation, indicating that this system also presents accuracy for pedicle screw placement without the need for intraoperative imaging.

When comparing a 3D machine-vision image-guided surgery (MvIGS) with traditional 2D fluoroscopy, Dorilio et al. (2021) [26] found that while operative times and pedicle screw placement accuracy were similar between the two techniques (96.6% for 2D and 94.2% for 3D), the MvIGS system lowered radiation exposure by eliminating the need for continuous intraoperative fluoroscopy through the use of optical topographical imaging. While not significant, both the radiation exposure time and dose were reduced in the 3D system, which enhances the idea that AR can further minimize radiation exposure while enhancing surgical efficiency. If later experiments provide a greater discrepancy favoring 3D techniques, this could provide a greater safety advantage for both patients and surgical staff. Based on a review by Sakai et al. [15], the studies referenced indicate that AR navigation systems reduced occupational radiation exposure to less than 0.01% of the reference level, while patient exposure was reduced to just 32% of the dose typically associated with alternative imaging techniques. Moreover, ARSN is a route to enhance minimally invasive spinal surgeries, as it not only maintains higher accuracy but also reduces the drawbacks associated with conventional imaging techniques. ARSN achieved comparable pedicle screw placement accuracy compared to traditional fluoroscopy (94% vs. 88%), while eliminating the need for intraoperative X-ray exposure [27]. Similarly, a study by Ramirez et al. [16] evaluated the effectiveness of reality technologies, such as xVision and Microsoft HoloLens, in spine surgery. HoloLens provided more spatial awareness and visibility, while xVision was able to reduce the time required for preoperative planning in minimally invasive surgeries, which also strengthens the idea that these technologies enhance overall efficiency for both the surgeon and the patient [28]. Table 1 displays the purposes of various AR technologies under development.

**Table 1:** Summary of Augmented Reality Technologies in Spine Surgery.

Study Reference	Purpose	AR Technology	Use in Spine Surgery	Key Findings
Babichenko et al. (2023) [29]	Review the development of AR in spinal procedures and discuss its potential.	Microsoft HoloLens	Evaluated cognitive load and performance during spine procedures using AR; also explored implications for spine surgical training	No significant difference in cognitive load between AR and non-AR use based on SURG-TLX; performance metrics were comparable.
Rush et al. (2022) [30]	Assess the impact of AR navigation on the accuracy and efficiency of preoperative planning and its impact on minimally invasive spinal procedures.	xVision	AR-assisted surgical navigation for percutaneous spine procedures	AR navigation using a virtual navigator (VN) was found to be accurate and potentially valuable for procedural planning. This shows applicability across various spine interventions.
Edström et al. (2020) [31]	Demonstrate the workflow and ease of an augmented reality surgical navigation (ARSN) system within a hybrid operating room environment.	ARSN system integrated with hybrid OR	Pedicle screw placement using intraoperative navigation	Microscope-based AR displays have been found to reduce attention shifts and maintain surgeon line-of-sight compared to conventional tools.
Schonfeld et al. (2021) [32]	Evaluate the ease and effectiveness of using AR goggles for real-time remote surgical guidance by overlaying visuals and audio into the surgeon’s field of view.	AR goggles, xVision	Provides a remote surgical assistance and intraoperative visualization	Surgeons reported that the AR goggles were comfortable to wear and made their workflow smoother; real-time 3D visualizations enhanced intraoperative decision-making and reduced complications.

User experience, learning curve, and OR integration

Setup and Intraoperative Use

Several factors should be considered when utilizing AR assistance, specifically the setup time, calibration of instrumentation, and the operative environment during surgical intervention. Devices such as the Microsoft HoloLens and xVision by Augmedics differ specifically in the nuances of setup; however, the approach is generally the same with AR across the board. For example, current devices typically require the integration of patient data, such as preoperative imaging, for accurate spatial computation and the display of various anatomical structures. Instrument calibration is usually needed for accurate tracking of the device during procedures as well. Concerning the setup environment, specifically for the Microsoft HoloLens 2 HMD, a variety of factors influence the device's performance in the OR, including light setup, assistant positioning and movement, HoloLens world/spatial anchor setup, and partition setup [33]. It was concluded that by standardizing and minimizing reflections through partitions, surgical personnel movement, and operative site illumination, the Microsoft HoloLens 2 HMD would perform similarly to that of one in a controlled laboratory setting [33]. Based on these findings, it is likely that similar spatial standardization can apply to other similar devices, such as the xVision, given the comparable need for spatially projecting 3D images in the surgical field and operating room. Regarding workflow, AR assistance has demonstrated a decrease in surgical duration for spinal procedures, such as posterior en bloc L1 corpectomy, reducing the duration to as little as six hours compared to the typical 11-14 hours [34]. With the continued reduction in surgical duration and standardization of AR environmental setup, the future use of AR can evolve to assist in more complex procedures, as well as contribute to the efficiency of case turnover [34-36].

Learning curve and surgeon perception

User education and feedback are also important aspects to consider. AR utilization with spinal procedures can provide trainees with additional visualization of complex anatomy and surgical precision that may otherwise prove challenging for those beginning their surgical training. In a study comparing the surgical outcomes of a minimally invasive transforaminal lumbar interbody fusion (MIS-TLIF) with and without the use of AR, there was found to be a significant increase in difficulty matching performance level (P = .019) as well as increased mental demand (P = .003) when AR was not used [35]. This exemplifies how the use of AR can help not only improve the efficiency of patient care but also reduce the mental burden amongst physicians for complex procedures. Positive feedback for the use of AR for training physicians has also been seen in other surgical procedures. When compared to traditional viewing monitors, Microsoft HoloLens HMD has shown improved procedural times (absolute difference, -73 seconds; 95% CI: -115 to -30; P = 0.0011) and Objective Structured Assessment of Technical Skills (OSATS) scores among procedures such as ureteroscopies (absolute difference, 4.1 points; 95% CI: 2.9-5.3; P < 0.0001) [36]. Overall, the use of AR across various specialties has proven to be a valuable and effective surgical aid for a range of procedures, applicable to diverse skill levels [37-39].

Barriers to adoption

While AR holds the potential to increase both precision and efficiency in spine surgery, its widespread adoption is hindered by several significant challenges. These barriers (technical, economic/institutional, and evidence gaps) continue to challenge the routine implementation of AR systems in the operating room.

Technical Barriers

Current AR platforms face technological limitations that hinder their routine intraoperative use. One primary challenge exists in depth perception, especially on AR platforms like Microsoft HoloLens 2 HMD, which project holographic 3D models into the user's field of view but typically rely on monoscopic displays. Unlike binocular vision systems, monocular AR fails to provide stereoscopic cues, which limits the surgeon's ability to judge depth accurately. This makes it difficult to assess the true trajectory of instruments relative to patient anatomy. A study by Uehira and Suzuki (2018) [37] demonstrated that while users can perceive depth using binocular disparity, the lack of realistic occlusion causes variability in perceived depth, especially at short distances. This becomes especially relevant in procedures such as pedicle screw insertion, where millimeter-scale precision is crucial. Katayama et al. (2023) [38] confirmed that this "depth perception problem" (DPP) persisted in surgical AR applications when holograms are projected onto a body surface. Although it can be partially mitigated by projecting holograms onto 3D models of deeper structures (i.e., bone) and varying the angle of observation, measurement errors of several millimeters still persist even with these adjustments. While refinements in display strategy can reduce this DPP, they may still fall short of the accuracy needed for intraoperative navigation.

Additionally, latency and overlay registration accuracy are well-documented clinical concerns for the implementation of AR. A review article by Shen et al. (2024) [5] found that even small mismatches between virtual overlays and patient anatomy, often caused by patient movement, anatomical distortion, or latency in tracking systems, can lead to significant errors. Target registration errors range from sub-millimeter to larger than 10 mm, depending on the method used (i.e., marker-based vs. surface-based registration). Holographic alignment is further compromised due to the limited field of view and fixed focal length of devices such as the Microsoft HoloLens 2 HMD. According to Creighton et al. (2020), these serve as primary contributors to misalignment and user-perceived distortion during orthopedic procedures. These spatial inaccuracies are further exacerbated by unstable tracking systems and a lack of integration into surgical operations, necessitating the development of improved sensor technology, faster registration systems, and more robust intraoperative calibration to maintain intraoperative accuracy [40-42].

Economic and Institutional Barriers

From an implementation perspective, the costs of these technologies remain a substantial hurdle. Hasan et al. (2023) noted that adopting an AR system in spinal surgery often requires a significant initial financial investment [43]. Advanced systems, like the Augmedics xVision, which integrate AR for intraoperative navigation, can range from $200,000 to $300,000. This includes expenses for the hardware, but additional training and onboarding costs must be accounted for. A lack of reimbursement pathways for AR interventions further exacerbates the economic burden and limits provision pathways within healthcare settings, especially for smaller institutions [44]. Additionally, these systems need to be integrated into existing surgical workflows, a complex process that requires not only initial additional training but also declines in efficiency while changes to established routines are being accommodated. These practical concerns, ranging from hardware costs to staff familiarity, can limit the scalability of AR technology.

Evidence gaps

While AR holds promise in transforming surgical training, substantial evidence gaps remain that undermine its validity and generalizability. In their systematic review, Suresh et al. (2022) analyzed 45 studies investigating the use of AR for effectiveness in surgical training across various specialties [10]. Only seven out of 29 AR platforms achieved a level of effectiveness, indicating observable changes in skill development with platform use; however, none demonstrated improved patient outcomes or systemic effects, such as cost savings and long-term skill retention. This underscores a lack of research connecting AR-based education to downstream or institutional benefits.

Additionally, the methodological quality of the studies varied. Fewer than one-third of the studies were randomized controlled trials, and only nine included more than 50 participants. The review applied Messick's five-parameter validity framework, assessing content, response processes, internal structure, relations to other variables, and consequences. While "content validity" was the most consistently assessed, other parameters, such as "response processes," "internal structure," and "consequences," were neglected in many of the studies. Many studies have quoted achieving vague concepts, such as "face validity," a subjective measure of perceived realism, without providing quantifiable or theory-based evidence. This approach skews the interpretation of the effectiveness of AR use in surgical training.

A large number of studies were observational or pilot studies with small sample sizes, short durations, and minimal follow-up, which prevents meaningful assessment of long-term skill retention or transferability to the clinical environment. Additionally, confounding factors such as users' learning curves with AR technology were rarely controlled. Improved performance may have stemmed from increased familiarity with the device rather than from the resulting educational value. Similarly, external learning (outside the simulation) was not accounted for in most protocols. Lastly, the participant pool was heavily skewed toward medical students, limiting insights into how experienced trainees or practicing surgeons perceive or benefit from AR [46-48].

These limitations highlight the need for more standardized and longitudinal research to establish the educational value and clinical impact of AR in clinical settings.

Summary of findings

AR has demonstrated its accuracy and ready-to-use nature to improve surgical and patient outcomes. This improved accuracy and guidance assistance for surgeons creates the added benefit of use in current surgical teams. There have been claims that AR enhances surgical advancements by enabling surgeons to visualize numerous internal structures through increased spatial awareness [46]. Overlaying information onto the surgical field improves precision in surgery. Through a systematic review, AR significantly reduced screw misplacement removal by 4.3%, compared to 8.9% with traditional methods. Li et al. (2023) demonstrated a 100% placement accuracy rate in screw placement through real-time navigation with an AR head-mounted device [47]. Managing complex surgical cases, AR systems also reduce operative time; one study reported a 20% reduction in operative time with AR assistance. Accurate instrument placement in spine surgery provides important background as aspects of malposition and misalignment in traditional surgeries are alleviated with AR [48]. Further explorations in AR-guided surgeries, utilizing tools like xVision by Augmedics, achieved screw accuracy with a 97.7% success rate, resulting in promising patient outcomes [49]. Other accurate procedures that achieved positive results included VisAR navigation, which had a 96% accuracy rate [50]. Spinal imaging plays an irreplaceable role, particularly in surgeries surrounding pedicle screw insertion, where even a slight error in screw placement can have catastrophic consequences, underscoring the clinical need for enhanced imaging and navigation technology. Fluoroscopy remains the conventional intraoperative imaging modality in minimally invasive spine surgery (MIS). Following these risks, including burns, cataracts, carcinogenesis, and hair loss, there remains a challenge to reduce fluoroscopy time and exposure.

AR technologies have already demonstrated measurable benefits in reducing radiation exposure. Recent pilot clinical trials compared the AR and AI-guided navigation using approaches such as cone beam computed tomography to standard fluoroscopy during percutaneous vertebroplasty (AR: 182.6 ± 106.7 mGy·cm² vs. FH: 367.8 ± 184.7 mGy·cm², p = 0.025) and fluoroscopy time (AR: 5.2 ± 2.6 s vs. FH: 10.4 ± 4.1 s, p = 0.005) [51]. These significant findings provide early support for the potential to alleviate the radiation burden, a central concern in image-guided spinal surgeries. Occupational exposure to ionizing radiation remains a substantial concern for spine and kyphoplasty cases. Current dosage models have demonstrated the quantitative risk that orthopedic surgeons face in the operating room. The standardized protective gear, a 0.5-mm lead-equivalent apron, allows for dosage levels of 5.1, 21, and 250 micro Sv [52]. Such levels of occupational exposure vary and are conventionally considered somewhat safe. Yet, new emerging technologies like AR can substantially reduce such exposure through the use of fluoroscopically assisted procedures and radiation protection. The introduction of imaging software, such as AR, would provide a protective means [50-59].

Future research needs

According to numerous reports, the current research is limited to small and single institutions, with limited generalizability due to the small cohort size. Current limitations hold to non-controlled and non-randomized studies, leading to possible bias in early outcomes [53].

Other pressing matters resurface, with a more cost-effective analysis and numerous return-on-investment studies for augmented platforms. Small pilot studies were conducted and analyzed, revealing a more favorable prognosis for patients [54]. Limitations and intricacies of AR systems range in price, cited as a range of $150,000 for AR platforms compared to conventional, with non-robotic alternatives at $400,000 to $1,000,000 [55]. This may add long-term value by reducing operative time, imaging costs, and many revision rates. Other prospective cases demonstrate substantial reductions in cost and revisions with AR systems called NaviVision (Vector Vision-BrainLAB AG, Feldkirchen, Germany) and Arcadis Orbic (Siemens Healthineers, Erlangen, Germany) [56]. Despite reasonably high equipment prices, the reduction to zero revisions and cost efficiency per case seems to outweigh these initial price setbacks.

Moreover, further research should investigate certain trade-offs in a clinical environment. Current AR systems can be adaptable for both open and minimally invasive techniques, suggesting potential cost savings through reduced dependency on hardware and ease of application. Other, more flexible options may even include an affordable and commercially available overlaying 3D product for surgical fields. Despite such promises, there is still more data to be aligned for larger-scale institutional investments. Another critical direction points to the expanding market and application of AR in surgery and medicine. The broader exploration and application of augmented systems beyond spine pedicle placement imply larger applications to other surgical fields, but there is a need to cement AR's role in surgery [57]. Current small studies suggest its application in various surgical fields; however, there is a need for a more established role of AR in surgery. Data are insufficient to discuss the full outcomes; however, there are promising outlooks for expanding new and improved surgical care. Simulation platforms for differing procedures, including spinal, have proven effective in improving residents' ability to perform lateral surgery [58]. Therefore, the curriculum served as a supplement to current AR/VR training. As described in the current literature, larger studies are needed to gather substantial quantitative data on the success of surgery and credentialing pathways for integration into current surgical use [59]. Expanding the applications of AR beyond spinal surgeries will unlock new visual flexibility for many trainees and surgeons, improving not only spinal care but also general surgical care.

## Conclusions

As futuristic implications expand within the field of medicine, the importance of implementing coursework surrounding AR tools in medical school, residency, and hospital fellowships is increasing. The importance of integrating AR into routine spine care helps expand surgical training programs, including cadaveric laboratories, guidelines from professional societies, and other credentialing pathways. Reports indicate that the use of simple or complex AR systems results in a 25% reduction in operative times compared to traditional methods. This tool serves to make the workflow more efficient and safer for patients and surgeons. These successful outcomes in spinal surgery will hinge on the need to standardize training. Descriptions of the surgeon's disorienting initial experience with AR prompt future discussion on how to better manage such sensory overload. This suggests a functional complement to the existing guidance-based systems.

Unlike robotic navigation, AR offers visual feedback. It is clear that as the field evolves, the ability to merge visual and surgical ergonomics can uniquely position AR as an enhancement, not a replacement, while preserving the fluency of traditional techniques. Thus, the importance of developing formal training pathways and simulation-based benchmarks ensures a safe integration into the surgical practice. Furthermore, future calls for meaningful implications for resident education and preoperative planning have yet to be demonstrated through large-scale studies that establish training.
